# Predictive Attentional Bias Modification Induces Stimulus-Evoked Attentional Bias for Threat

**DOI:** 10.5964/ejop.v15i3.1633

**Published:** 2019-09-27

**Authors:** Thomas E. Gladwin, Martin Möbius, Eni S. Becker

**Affiliations:** a Institute of Education, Health and Social Sciences, University of Chichester, Chichester, United Kingdom; b Behavioural Science Institute, Radboud University Nijmegen, Nijmegen, The Netherlands; Webster University Geneva, Geneva, Switzerland

**Keywords:** attentional bias modification, attention, threat, predictive cues, attention bias

## Abstract

Attentional Bias Modification (ABM) aims to modulate attentional biases, but questions remain about its efficacy and there may be new variants yet to explore. The current study tested effects of a novel version of ABM, predictive ABM (predABM), using visually neutral cues predicting the locations of future threatening and neutral stimuli that had a chance of appearing after a delay. Such effects could also help understand anticipatory attentional biases measured using cued Visual Probe Tasks. One hundred and two participants completed the experiment online. We tested whether training Towards Threat versus Away from Threat contingencies on the predABM would cause subsequent attentional biases towards versus away from threat versus neutral stimuli, respectively. Participants were randomly assigned and compared on attentional bias measured via a post-training Dot-Probe task. A significant difference was found between the attentional bias in the Towards Threat versus Away from Threat group. The training contingencies induced effects on bias in the expected direction, although the bias in each group separately did not reach significance. Stronger effects may require multiple training sessions. Nevertheless, the primary test confirmed the hypothesis, showing that the predABM is a potentially interesting variant of ABM. Theoretically, the results show that automatization may involve the process of selecting the outcome of a cognitive response, rather than a simple stimulus-response association. Training based on contingencies involving predicted stimuli affect subsequent attentional measures and could be of interest in future clinical studies.

Attentional biases are automatic processes that influence the selection of information for further processing (Cisler & Koster, 2010; Kane & Engle, 2003). Spatial attentional biases, involving the direction of attention to the location of salient cues, can be measured via dot-probe or visual probe tasks (VPTs) (MacLeod, Mathews, & Tata, 1986). Faster responses to probe stimuli appearing at the location of certain cue categories provides an implicit measure of bias (Cisler & Koster, 2010; Mogg & Bradley, 2016; Notebaert, Crombez, Van Damme, De Houwer, & Theeuwes, 2011). An interesting application of research into attentional biases is their use in training paradigms, termed Attentional Bias Modification, ABM (MacLeod & Mathews, 2012). ABM aims to reverse a putatively harmful attentional bias in order to change associated behavior or symptoms, such as spider phobia (Luo et al., 2015), depression (Ferrari, Möbius, van Opdorp, Becker, & Rinck, 2016; Wells & Beevers, 2010), addiction (Schoenmakers et al., 2010) and PTSD (Kuckertz et al., 2014). Opinions are strongly divided, however, on the efficacy of ABM, and it appears that, at the least, its efficacy is conditional on moderating factors (Clarke, Notebaert, & MacLeod, 2014; Cristea, Kok, & Cuijpers, 2015; Gladwin, Wiers, & Wiers, 2016).

Some recent studies have raised interesting possibilities potentially relevant to ABM. First, cued Visual Probe Tasks (cVPTs) have been developed to the aim of studying outcome-related attentional biases (Gladwin, 2016). In the cVPT, instead of presenting salient cues and determining how they affect attention, visually neutral predictive cues are used. The predictive value is caused by Picture trials, in which the predictive cues are replaced by an actual exemplar from the category associated with them, for example, threat versus neutral. Assessment of the bias is based on Probe trials, on which instead of the exemplar a probe stimulus requiring a response is presented. Thus, performance is not dependent on a given trial’s specific exemplars, but on the predicted categories of stimuli that could have been presented. Possibly partly due to this removal of a source of variability, the cVPT has been found to have relatively good reliability (Gladwin, 2018; Gladwin, Möbius, Mcloughlin, & Tyndall, 2018). A theoretical question is what is causing the bias. The task design implies that the cues serve as predictive stimuli for possible outcomes, or as a kind of prime (Kristjánsson & Ásgeirsson, 2019), due to some form of learning process (Failing & Theeuwes, 2018). However, it is conceivable that the visual features of the cues themselves acquire salience, as opposed to the theoretically motivating idea that predictive mechanisms determining the outcome of attentional shifting would result in the bias (Gladwin & Figner, 2014; Gladwin, Figner, Crone, & Wiers, 2011). Using a cVPT as an ABM task could provide evidence to help address this issue: if using a cVPT to train participants’ attention towards or away from an outcome indeed results in a bias involving the stimulus categories, rather than the specific cues used during training, this would suggest that the cVPT involves outcome-related processes rather than cue-specific learning.

Second, positive effects have been reported of what would usually be considered control conditions of ABM, in which no specific bias was induced but probes had a random relationship with emotional cues (Badura-Brack et al., 2015; Gladwin, 2017; Khanna et al., 2015). It has been suggested that whether a training variant makes emotional cues relevant or irrelevant to the training task may be an important factor in ABM (Gladwin, 2017). In usual sham conditions, emotional cues are irrelevant to the task and thus participants could be learning to ignore such stimuli when confronted by them. For instance, in a control condition of a training based on the Dot-Probe task, the location of emotional cues is non-predictive of the location of probe stimuli. In active training conditions, while the aim is to affect the direction of attentional biases, it is also usually the case that emotional information is relevant. In the Dot-Probe example, if probe locations are contingent on the location of emotional cues, then that makes those cues relevant. This could induce a “salience side-effect” in some designs: Participants may be learning to pay attention to the location of task-relevant emotional stimuli, even if the direction of attentional shifting is away from them. This could add noise and complexity to results, with different processes being affected in uncontrolled ways during training. In line with the idea that salience is an important factor in training, Approach-Avoidance Retraining for alcohol addiction reduced amygdala reactivity to alcohol stimuli (Wiers et al., 2015), which was interpreted as a neural signature of salience reduction.

The goal of the current study was to explore a novel form of ABM hypothesized to avoid this salience side-effect, which simultaneously may help understand the nature of the anticipatory spatial attentional bias. A training version of the cued Visual Probe Task was used, in which the probability of the location of probes relative to the outcome of cues is manipulated. This was termed predictive Attentional Bias Modification (predABM). To test whether this kind of predictive-cue training would affect attentional bias towards or away from actually presented emotional stimuli, a Towards Threat training condition and an Away from Threat training condition were compared using a normal Dot-Probe task post-training. As the delay between emotional cues and probe stimuli in Dot-Probe tasks is known to be potentially time-dependent (Mogg & Bradley, 2006; Noël et al., 2006), multiple cue-stimulus intervals were used. Note that due to the experimentally controlled random allocation of participants to groups, this post-only design allows valid statistical inference to be done: Statistically significant differences between groups on the post-test measures can be interpreted as an effect of training, with only the usual possibility of a false positive (which would also be present when analyzing difference scores). Beyond this basic point on the validity of randomized post-only designs, there are advantages and disadvantages to using a post-only versus pre-post design discussed further in the Discussion section. We hypothesized that training to shift attention towards versus away from the location of predicted upcoming threatening facial stimuli would affect attentional bias towards or away from such stimuli on the post-test stimulus-evoked attentional bias.

## Method

### Participants

Participants were recruited from a student population and received study credits for completing the study. Participants gave informed consent and the study was approved by the local ethics review board. The study was performed online. One hundred and two participants completed the experiment (88% female, 22 % male; mean age 20, *SD* = 0.29). The study was performed fully online.

### Materials

#### Questionnaires

The following questionnaires were used as the set of covariates to reduce training-unrelated variance on the post-test Dot-Probe task. The aim was to use a range of questionnaires concerning individual differences, which could affect attentional biases involving threat: Anxiety, post-traumatic stress disorder, depression, and aggression. The questionnaire on depression was unfortunately lost due to a technical error. Note that because the between-subject factor of training was randomly assigned it was stochastically independent from the covariates, providing an appropriate situation for the use of analysis of covariance.

The TSQ (Brewin et al., 2002) was used to estimate the presence of post-traumatic stress symptoms. Participants were asked to indicate for each of the 10 items, whether they experienced the described symptom (at least twice) in the past week or not. The total score ranges between 0 and 10, while higher scores represent the presence of more PTSD symptoms.

To assess an individual’s disposition to aggressive behavior we used the Buss-Perry Aggression Questionnaire (Buss & Perry, 1992). This questionnaire consists of four subscales; I) physical aggression, II) verbal aggression, III) anger, IV) hostility. On 29 items, participants had to indicate how characteristic each of the described behaviors was in describing them (1 = *totally uncharacteristic*, 5 = *totally characteristic*), with higher scores reflecting greater disposition for aggressive behavior.

The short version of the STAI, STAI-6 (Marteau & Bekker, 1992) was used to measure changes in individual state anxiety. This scale comprises six statements to be rated on a 4-point Likert scale (1 = *not at all*, 4 = *very much*). We calculated a weighted sum score in which responses on the three items involving positive feelings were multiplied by -1. Higher sum scores represent higher state anxiety levels.

#### The predABM Training Task

The predABM task was administered to modify attentional processing to threatening stimuli. The faces of 16 characters, each with an angry and a neutral expression, from the BESST (Thoma, Soria Bauser, & Suchan, 2013) were used. The task consisted of 24 blocks of 24 trials each. All trials started with a fixation cross (300, 400, or 500 ms) followed by the appearance of two initially neutral cues one above the other, each of which consisted of a horizontal row of five differently colored typographical symbols (e.g., five blue crosses). After every eight blocks, a different pair of cues was used. The aim of this was to reduce the chance that participants would only learn a contingency involving a particular pair of cue-stimuli, rather than the outcome-contingency which was consistent over the varying cue pairs. The cues were presented for a CSI of 200 or 1,200 ms, with equal probability, so as not to induce CSI-related differences with the dot-probe assessment. The essential feature of the task is that there were two trial-types, which were presented with equal probability; On half of the trials (“picture trials”), one of the cues (randomized per subject) was replaced by a picture of an angry face, and the other by a picture of a neutral face. On the other half of the trials (“probe trials”), the trial continued as in a normal dot-probe task, with the probe-distractor pair replacing the cues. The probe stimulus was an arrow-like symbol pointing to the left < or right >. The distractor stimulus was a /\ or \/. The distractors were used to make it more difficult to respond without focusing attention on the correct location, since they were visually similar to the probe stimuli. Participants were instructed to press the corresponding left or right key (F or J on the keyboard) within 800 ms. Correct answers were followed by the word “Good” (“Goed,” in Dutch) in green, while incorrect answers were followed by the word “Wrong” (“Fout,” in Dutch) in red. When no response was registered the term “Too late” (“Te laat,” in Dutch) was presented in red. This feedback remained on the screen for 500 ms. Essentially, the picture trials were designed to train an association between cues and the possible appearance of angry versus neutral pictures at their location, and the probe trials provided an assessment of effects of that association.

In both groups, cues consistently predicted the locations of threat and neutral stimuli. They only differed in their relationship to where probe stimuli would appear. In the Towards Threat group, 90% of probes appeared at the location where an angry face was predicted to appear. In the Away from Threat group, 90% of probes appeared at the location where a neutral face was predicted to appear.

#### Dot-probe Task

For the dot-probe task a subset of 16 faces from the BESST was used, different from the subset used during training. The task consisted of four blocks of 24 trials. Each trial started with a fixation cross (300, 400, or 500 ms) followed by the presentation of an angry and a neutral face, one above the other, for 200 or 1,200 ms, with equal probability. Trials then continued precisely as in the probe trials in the predABM task described above: a probe-distractor pair replaced the cues, to which participants had 800 ms to respond, followed by feedback.

### Procedure

Individuals who chose to participate were guided to the web page for the experiment via a Sona Systems participant pool. They viewed a page with participant information and gave informed consent via a button to continue. The next page briefly repeated the most essential information and gave tips for correct performance of the tasks, for example, turning off phones, maximizing the browser window, and closing other programs and browser tabs. Participants filled in questionnaires and then performed the predABM and Dot-Probe task. Participants were assigned to a training condition at random. In the same session, participants also completed questionnaires and tasks unrelated to the current study.

### Statistical Analyses

First, within-subject repeated measures ANOVAs were performed per training group to determine whether each training condition had the expected effects on behavior during training. For each training condition (Towards Threat condition and Away from Threat) it was tested whether the respective bias was induced during the training (within-subject factors Probe Location and CSI), although of course these tests do not indicate whether such biases involved the predicted outcome as opposed to the initially visually neutral cues. Probe Location refers to whether the probe appeared at the location of the Threat or Neutral cue. Dependent variables were median RT (the median was used to reduce the impact of outliers, without needing to specify an arbitrary cut-off for outliers as would be necessary with the mean) and mean accuracy, calculated for all probe trials. The questionnaire data (i.e., age, sex, Buss-Perry subscale scores, TSQ, and STAI-6) were included as covariates.

Second, and most essentially, effects of the attentional manipulation on the Dot-Probe task were tested using mixed design ANCOVAs, with within-subject factors Probe Location (Neutral, Threat) and CSI (200 ms, 1,200 ms) and between-subject factor Training condition. The questionnaire scores were included as covariates. It was tested whether the training conditions (Toward Threat vs. Away from Threat) induced reversed attentional biases on the Dot-Probe task. Dependent variables were median reaction time and mean accuracy.

## Results

Table 1 shows descriptive statistics for the questionnaire data. Fifty-four participants were assigned to the Away from Threat group and 48 to the Towards Threat group.

**Table 1:** Demographics and Questionnaire Data

Score	Away from Threat	Towards Threat
Sex	78%	92%
Age	19.8 (2.06)	19.5 (1.44)
BP—Physical Aggression	19.6 (5.66)	16.6 (4.99)
BP—Verbal Aggression	17.4 (3.76)	15.6 (2.82)
BP—Anger	16.9 (5.45)	16.9 (6.38)
BP—Hostility	20.1 (8.15)	17.9 (8.1)
Trauma screening questionnaire	3.02 (2.94)	3.04 (2.8)
STAI, pre-training	-4.11 (2.93)	-3.83 (3.3)
STAI, post-training	-3.15 (3.11)	-3.21 (3.05)

*Note.* The values are percentages (for Sex, percentage female) and mean values, with standard deviations in parentheses. BP represents the Buss-Perry questionnaire. The STAI scores were calculated as the sum of negative minus the sum of positive items.

### Performance Data on the predABM During Training Conditions

Table 2 shows descriptive statistics for the predABM. In the Away From Threat group, responses to probes on Threat locations were slower than responses to probes on Neutral locations, *F*(1, 53) = 5.67, *p* = .021, $\eta_{p}^{2}$ = .097. An effect of CSI was found, *F*(1, 53) = 66.53, *p* < .001, $\eta_{p}^{2}$ = .56, due to slower responses at the long (1,200 ms) versus short (200 ms) CSI. In the Towards Threat group, responses to probes on Threat locations were faster than responses to probes on Neutral locations, *F*(1, 47) = 4.55, *p* = .038, $\eta_{p}^{2}$ = .09. An effect of CSI was found, *F*(1, 47) = 24.68, *p* < .001, $\eta_{p}^{2}$ = .34, due to slower responses at the long versus short CSI.

**Table 2:** Performance Data on the predABM

Probe Location	CSI	Away From Threat	Towards Threat
**Reaction time [ms]**			
Neutral	200 ms	528 (51)	536 (77)
Neutral	1,200 ms	558 (62)	553 (58)
Angry	200 ms	537 (69)	521 (52)
Angry	1,200 ms	573 (75)	551 (58)
**Accuracy**			
Neutral	200 ms	0.97 (0.059)	0.96 (0.066)
Neutral	1,200 ms	0.96 (0.078)	0.97 (0.045)
Angry	200 ms	0.97 (0.042)	0.98 (0.017)
Angry	1,200 ms	0.97 (0.049)	0.98 (0.016)

*Note.* Means and standard deviations for reaction time and accuracy on the predABM task. Measure refers to performance measure, that is, reaction time and accuracy. Probe location refers to the location where the probe stimulus appeared: The location of the cue where Neutral faces versus the cue where Angry faces would appear on non-probe trials. CSI refers to Cue-Stimulus Interval, the delay between cue presentation and probe presentation.

### Training effects on the Dot-Probe Task

Descriptive statistics for the Dot-Probe Task are shown in Table 3.

**Table 3:** Performance Data on the Dot-Probe task

Probe Location	CSI	Away From Threat	Towards Threat
**Reaction time [ms]**			
Neutral	200 ms	505 (56)	502 (55)
Neutral	1,200 ms	516 (53)	511 (50)
Angry	200 ms	504 (58)	497 (53)
Angry	1,200 ms	523 (56)	511 (52)
**Accuracy**			
Neutral	200 ms	0.96 (0.051)	0.95 (0.047)
Neutral	1,200 ms	0.97 (0.052)	0.96 (0.042)
Angry	200 ms	0.96 (0.048)	0.97 (0.045)
Angry	1,200 ms	0.97 (0.046)	0.97 (0.04)

*Note.* Means and standard deviations for reaction time and accuracy on the Dot-Probe task. Measure refers to the performance measures reaction time and accuracy. Probe location refers to the location where the probe stimulus appeared: The location of the cue where Neutral faces versus the cue where Angry faces would appear on non-probe trials. CSI refers to Cue-Stimulus Interval, the delay between cue presentation and probe presentation. Away from Threat and Toward Threat refer to the training conditions.

**Figure 1:**
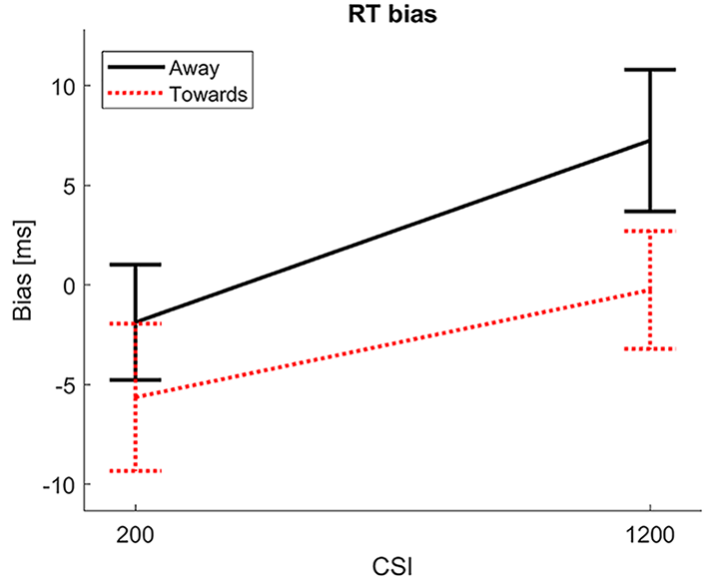
Post-training RT bias per training group. *Note.* The Figure shows the attentional bias, RT for Angry minus RT for Neutral, following the Towards Threat and Away from Threat training. The groups showed a relative shift in bias as expected.

On RT, the hypothesized effect was found of Group × Probe Location, *F*(1, 91) = 4.75, *p* = .033, $\eta_{p}^{2}$ = .05, shown in Figure 1.

The Towards Threat group had a bias towards threat relative to the Away from Threat group. The direction of the effect of Probe Location was reversed as expected between the groups, with shorter RTs on the Neutral than on the Threat location in the Away from Threat group, and shorter RTs on the Threat than on the Neutral location in the Towards Threat group. We do note that the magnitudes of the biases were small however, and the main effect of Probe Location did not reach significance in either group separately, despite the significant Group × Probe Location interaction. Further, an effect of CSI was found, *F*(1, 91) = 7.95, *p* = .0060, $\eta_{p}^{2}$ = .08, due to slower responses at the long versus short CSI. No effects on accuracy were found.

## Discussion

The aim of the current study was to provide a first test of the effects of predABM, a novel version of ABM using predictive cues. Rather than being trained to direct attention towards or away from threatening stimuli, participants were trained to direct attention towards or away from locations based on cues predicting where a threatening stimulus could appear. Thus, the training did not involve a direct stimulus-response association between stimuli in the threat category and attentional shifting, a feature of usual training tasks that could result in unexpected effects involving salience. The question was whether training using predictive cues would be able to affect stimulus-evoked attentional bias.

Performance data during training blocks showed that participants responded to the outcome-based task contingencies as expected. Responses were faster to probes appearing at the trained location. Note that this could reflect either an association involving the specific predictive cues or an association involving the stimulus category predicted by the cues—initial cVPT studies (Gladwin, 2016; Gladwin et al., 2018; Gladwin & Vink, 2018) were not able to distinguish between such possibilities concerning underlying mechanisms. Whether the latter, outcome-focused kind of association occurred was tested by the post-training generalization to the Dot-Probe task described below. Training effects were in fact found on the Dot-Probe task presented after the training. The Towards Threat group and Away from Threat group showed the expected relative decrease and increase, respectively, in reaction time for probes on Angry versus Neutral locations. Thus, the attentional response to emotional stimuli was changed via the stimulus categories of the outcomes of the predictive cues during training. Essentially, therefore, it was not the case that participants only learned to shift attention towards or away from the specific predictive cues. The results show that training involved the stimulus categories that were predicted by the initially visually neutral cues, even though the emotional stimuli never appeared on the same trial as the probe stimuli.

The results thus provide first support for the potential use of predABM. Although concern for salience side-effects in ABM, due to the informativeness of emotional cues, is as yet a recent development, the predABM provides a method that appears to be able to address this potential problem. However, we note that the potential training value of using an anticipatory attentional bias based on upcoming emotional stimuli, rather than responses to already-presented emotional stimuli, does not only depend on the salience side-effect. Anticipatory or preparatory processes related to emotional stimuli could be an interesting target for training in themselves, as this may have different effects from ABM involving stimulus-evoked processes. Further, a feature of predictive cues is that a wide range of possible stimuli can be associated with single conditioned cue. An interesting direction for future research is whether this may improve generalization to other stimuli, since attention is directed towards an abstract category rather than a specific set of stimuli.

A limitation of the current study is that only a single session was used, while effects of multiple sessions are likely most relevant for potential clinical applications and could provide larger effect sizes. However, the current results provide a proof-of-principle that the outcome-focused cued training task was able to change attentional processes related to the predicted stimulus category. A further limitation is that the population involved a sample of students. Patient groups are clearly an important target population, and it remains to be determined whether non-student samples respond to the training contingencies in the same way. A concern with training methods, especially for future use in clinical populations, is their impact on patients. The current study was also limited in its use of computer-generated angry faces as emotional stimuli: It cannot be assumed that the effects will generalize to other stimulus categories. Different results might be obtained in future research with, for example, stimuli representing physical threat, or verbal stimuli designed to evoke shame or guilt. Concerning the design, only a post-training assessment task for attentional bias was used, similarly to analyses involving post-training effects in previous studies (e.g., Gladwin et al., 2015). We note that, while pre-post designs have the advantage of providing a pre-training measurement, the logic of a post-training experimental design, with random assignment, is equally valid statistically: The chance of the groups having training-independent differences in attentional bias at post-test at random is the same as the chance of groups having training-independent *changes* in attentional bias from pre- to post-test at random. Further, a post-test design avoids test-retest effects, which could be a source of noise. There may also be theoretical reasons to expect effects to be caused on post-test states, rather than on pre-post shifts. Thus, while arguments can be made for either design, there is no reason to consider the lack of a pre-test a particular threat to the validity of conclusions drawn from the results. Finally, more work is needed to further explore the nature of training effects. Psychophysiology or neuroimaging methods could help test hypotheses on which underlying processes are affected, such as cue reactivity measures indicating changes in salience (Wiers et al., 2015) or attentional control (Eldar & Bar-Haim, 2010).

In conclusion, training to shift attention based on the expected stimulus-locations induces changes in attentional biases. The use of predictive cues in training may open interesting directions for further study.
